# Feasibility of same-day discharge after transcatheter aortic valve replacement: the North Shore Day Stay pathway

**DOI:** 10.1007/s00380-025-02598-4

**Published:** 2025-09-22

**Authors:** Karan Rao, Princess Neila Litkouhi, Alexandra Baer, Peter Hansen, Ravinay Bhindi

**Affiliations:** 1 Department of Cardiology, Royal North Shore Hospital, St. Leonards, Reserve Road, Sydney, 2065 Australia; 2 University of Sydney, Sydney, Australia; 3 Department of Cardiology, North Shore Private Hospital, Sydney, Australia

**Keywords:** Aortic valve replacement, Same-day discharge

## Abstract

Transcatheter aortic valve replacement (TAVR) is an established treatment for patients with severe symptomatic aortic stenosis but expanding indications have increased strain on hospital resources. Several studies assessed same-day discharge (SDD) after TAVR during the COVID-19 pandemic and showed it to be safe in well-selected, low-risk patients. However, more studies are warranted, with no studies in an Australian population and minimal data on self-expanding valves. Patients undergoing consecutive, transfemoral TAVR procedures at two large-volume centres in Sydney, Australia between 2021 and 2023 were prospectively recruited to the CONDUCT–TAVI study cohort. A locally derived clinical pathway (‘The North Shore Pathway’) was retrospectively applied to identify which patients would have been suitable for SDD. In-hospital and 30-day outcomes were compared between SDD patients and the remaining, ineligible patients (standard discharge cohort). Of 182 patients, 20 (11.0%) met SDD criteria. The total cohort received both self-expanding (67.3%) and balloon-expandable valves (32.7%). The SDD cohort had a higher proportion of females (55.0% vs. 21.5%, *p* = 0.04) but was otherwise comparable in baseline and procedural characteristics. No significant differences were found in hospital or 30-day outcomes. One SDD patient was readmitted with complete heart block requiring pacemaker implantation (day 20), and two patients had non-cardiovascular readmissions. No other adverse outcomes occurred in the SDD cohort. The present study suggests SDD after TAVR is feasible in both balloon-expandable and self-expanding cohorts. The study also supports prospective validation of the North Shore Day Stay pathway as a tool to safely identify low-risk patients that are suitable for SDD after TAVR.

## Introduction

Transcatheter aortic valve replacement (TAVR) is an effective treatment for patients with symptomatic severe aortic stenosis. The prevalence of aortic stenosis is expected to more than double by 2050 [1], placing increased strain on already stretched hospital systems and risking delays to patients accessing definitive treatment. Consequently, there is an urgent need to streamline TAVR without compromising patient safety. Although median length of stay has steadily declined since 2021 [2], bed availability particularly in the public sector remains challenging.

A proposed strategy for improved bed utilisation is same-day and next-day discharge in highly selective, low-risk TAVR patients. In 2019, The Vancouver 3 M study demonstrated that next-day discharge (NDD) after TAVR could safely occur in 80% of a highly selective cohort, with up to 20% cost reduction [3]. More recently, same-day discharge (SDD) has been trialled in a number of centres in North America and the United Kingdom and showed comparable outcomes between SDD and NDD in appropriately selected elective TAVR patients [4, 5]. Early discharge may also reduce the risk of nosocomial complications, such as deconditioning, delirium and infection. However, more studies and the development of a standardised discharge protocol are needed to support a broader implementation of SDD.

Self-expanding valves, historically a strong independent predictor of high-grade atrioventricular block (HGAVB) after TAVR [6], are under-represented in current SDD studies, which have predominantly focused on balloon-expandable valves. However, newer-generation self-expanding valves have demonstrated comparable pacemaker outcomes across all valve types [7], paving way for studies to assess the feasibility of SDD in self-expanding valves.

This study aims to apply a locally derived SDD pathway to evaluate the safety and feasibility of SDD in a low-risk Australian TAVR cohort receiving both self-expanding and balloon-expandable valves.

## Materials and methods

### Study design and population

Retrospective analysis was performed on patients originally enrolled into a prospective observational study, CONDUCT–TAVI (ACTRN1261001700820). CONDUCT–TAVI recruited consecutive patients undergoing TAVR at two high-volume centres in Sydney, Australia, between October 2021 and December 2023. Patients were excluded if they had a prior permanent pacemaker (PPM) or aortic valve surgery. Furthermore, if a PPM was not required post-procedure, all patients received an implantable loop recorder prior to discharge. Further detailed information on the CONDUCT–TAVI protocol is publicly available [8]. The protocol has been approved by the Northern Sydney Local Health District Ethics Committee.

The CONDUCT–TAVI cohort was chosen for this study due to the availability of extended rhythm monitoring, which was deemed necessary due to the inclusion of patients receiving self-expanding valves.

### TAVR procedure

All patients underwent “*minimalist*” TAVR, which was performed under conscious sedation via the transfemoral route. Secondary access was either transradial or transfemoral, and single transfemoral transvenous access was obtained for a multipolar electrophysiology catheter for immediate pre and post TAVR targeted electrophysiology study, as mandated by the CONDUCT–TAVI research protocol. Rapid ventricular pacing was performed over the left ventricular wire in all cases. Cerebral protection devices were not used.

Pre-dilatation, valve type and size, as well as post-dilatation was at the implanters’ discretion. All patients were discharged to a coronary care unit with continuous cardiac monitoring for a minimum of 24 h. All patients had an immediate post-procedure, 4-h and 24-h ECG, and underwent standard pre and post procedure echocardiography (day 0 and day 1).

### North Shore Day Stay clinical pathway

**Fig. 1:**
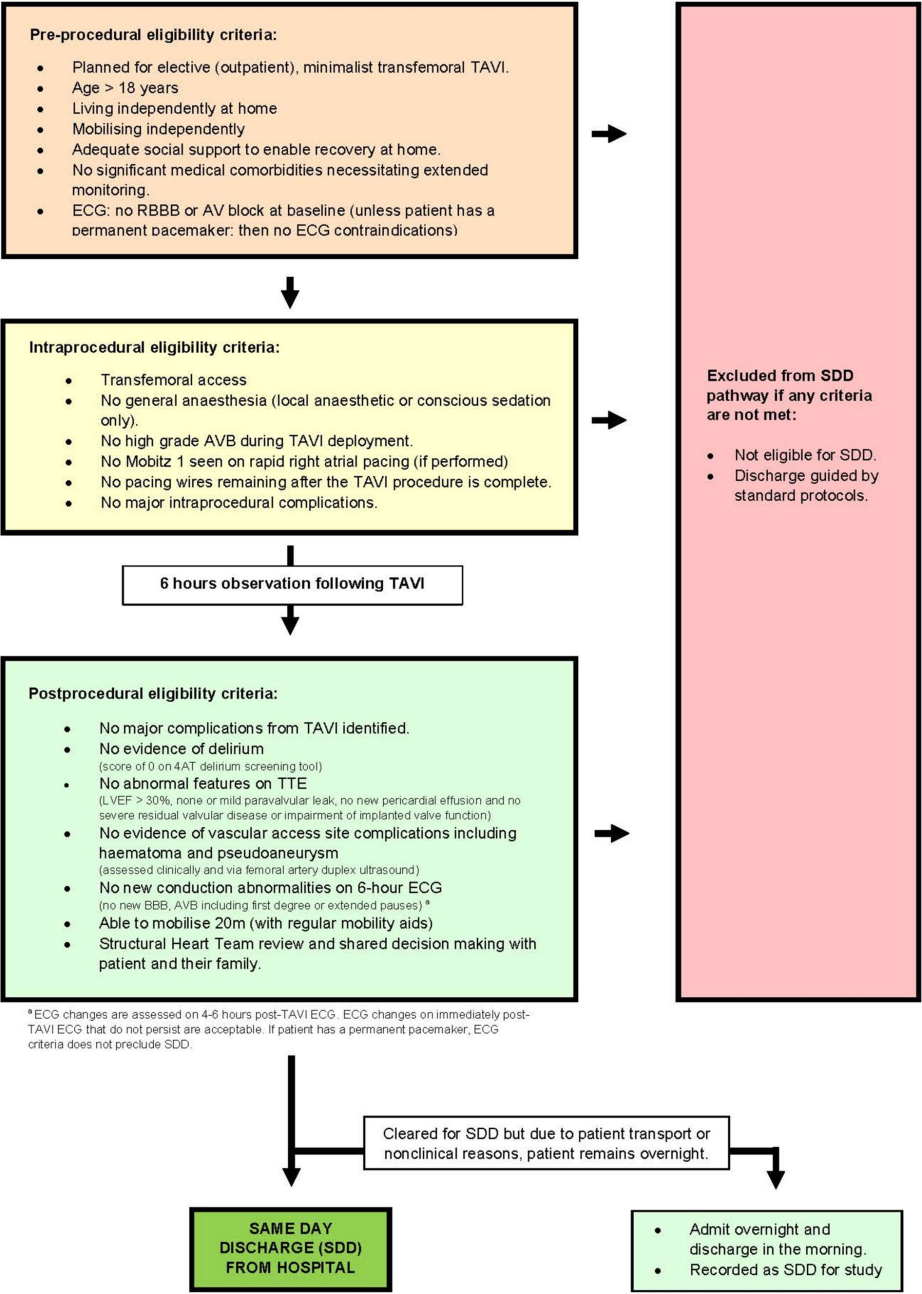
NORTH SHORE DAY STAY TAVI clinical pathway

The North Shore Day Stay clinical pathway (the ‘North Shore Pathway’) was adapted from previously published protocols [4, 5, 9]. It was retrospectively applied to all patients enrolled in CONDUCT–TAVI to assess theoretical suitability for SDD. The protocol included comprehensive preprocedural, procedural and postprocedural criteria, as illustrated in Fig. 1.

Preprocedural inclusion criteria included non-urgent, elective TAVR, age ≥ 18 years, mobilising and living independently, adequate social supports to facilitate recovery at home and no significant medical comorbidities that necessitated additional in-hospital monitoring, such as end-stage renal disease or advanced dementia. Other exclusion criteria were all conduction-related and derived from the baseline ECG. These included a pre-existing right bundle branch block (RBBB) or atrioventricular block (AVB) (first, second or third degree). Whilst the North Shore pathway provides an exception for patients with a prior PPM from conduction-related exclusion criteria, this was not applicable in our cohort.

The procedural requirements were the use of conscious sedation (no general anaesthesia), and the absence of any HGAVB during valve deployment. Furthermore, patients were required to have no recorded intraprocedural complications as per the Valve Academic Research Constorium-3 (VARC-3) criteria [10], and temporary pacing wires were required to be removed prior to the end of the case.

Postprocedural requirements were assessed at 4–6-h post-procedure and included: (1) no evidence of delirium, (2) no evidence of vascular access site complications, such as haematoma or pseudoaneurysm, (3) no abnormal transthoracic echocardiogram features (LVEF > 30%, none or mild paravalvular leak, no new pericardial effusion and functioning implanted valve), (4) no new conduction abnormalities (new AVB, including first degree block, or any new bundle branch block) unless patient had a permanent pacemaker, (5) ability to mobilise 20 m (assessed with the patient’s regular mobility aids) and (6) multidisciplinary team agreement of suitability for SDD.

Given the retrospective nature of this study, evidence of delirium and vascular access site complications were determined by clinical assessment which was established from medical documentation. Other studies investigating SDD in TAVR patients have used clinical examination to assess for delirium and vascular access site complications [4, 5]. Similarly, mobility criteria were also assessed using medical, nursing, or allied health clinical documentation. Patients that were documented to have independently mobilised, for example, to the bathroom outside the room, were considered to have satisfied mobility criteria. Patients that remained on bedrest, required use of a bedpan, or had no explicit mention of their mobility, were considered not to have satisfied mobility criteria.

Assessment for SDD eligibility aimed to replicate real-time decision making by restricting investigators to information only available at each timepoint (pre-TAVR, immediately post-TAVR and 6-h post-TAVR), and blinding to all subsequent in-hospital events and 30-day events.

Patients who met all preprocedural, procedural and postprocedural criteria outlined in the clinical pathway were allocated to the SDD cohort. Patients who did not satisfy one or more criteria were allocated to the standard discharge cohort.

### Outcome measures

Detailed patient demographics were obtained and compared between SDD and standard discharge groups. This included age, sex, body mass index and the Society of Thoracic Surgeons–Predicted Risk of Mortality (STS–PROM) score, along with medical comorbidities, prior cardiac procedures, and echocardiographic data. Baseline electrocardiography was also recorded.

Outcomes measured were in-hospital and 30-day post-discharge events, which were defined as per the VARC-3 criteria [10]. The 30-day post-discharge events were defined as occurring following discharge and up to 30 days after their procedure, and included death, hospital re-admission (any cause, cardiovascular and non-cardiovascular), timing of readmission, and detailed post-discharge events, including new permanent pacemaker implantation (PPMI), stroke, vascular access complications, bleeding, delirium and myocardial infarction.

As an additional timepoint, outcomes were recorded in the SDD cohort at the median discharge day of the standard cohort. This aimed to assess whether patients selected for SDD developed complications during the period they would have otherwise remained in hospital, to evaluate the clinical benefit of extended inpatient monitoring in this cohort.

### Statistical analysis

Statistical analysis was performed using IBM SPSS Version 29.0. Categorical variables were assessed via Fisher’s exact test, or a chi-square test. Continuous variables were reported as a mean if normally distributed and compared using a *T* test, or via a median if not normally distributed, and assessed via a Mann–Whitney *U* statistical test. A two-tailed *p* value of < 0.05 was considered statistically significant.

## Results

### Cohort demographics and procedural characteristics

182 patients enrolled in CONDUCT–TAVI were screened for SDD suitability. The mean age of the cohort was 82.0 (± 6.4) years, whilst 120 (65.9%) were male. Median STS Score was 2.6 (± 1.4) corresponding to low-surgical risk. 56 (30.8%) patients had a history of prior atrial fibrillation, whilst on pre-TAVR ECG, 47 (26.0%) had first degree AVB, 31 (17.0%) had RBBB, and 12 (6.6%) had left bundle branch block (LBBB). All TAVR cases were electively performed under conscious sedation, via the transfemoral route. Self-expanding valves were the predominant valve type used (65.4%), with balloon-expandable valves used for the remainder (34.6%).

### Predictors of SDD

**Fig. 2:**
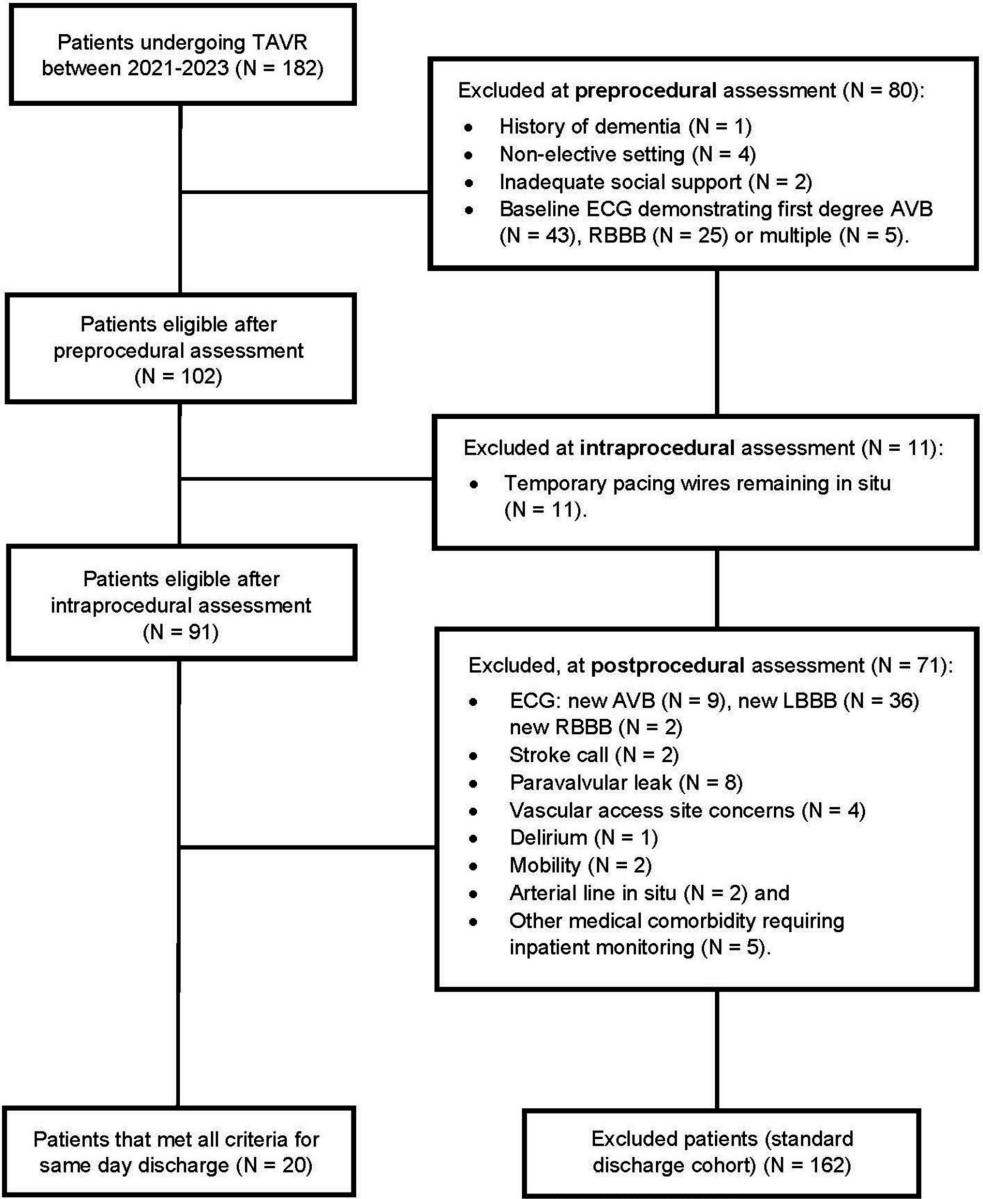
Study Flowchart

After application of the North Shore pathway, a total of 20 (11.0%) patients were deemed suitable for SDD, whilst 162 (89.0%) were excluded and remained on the standard discharge pathway. Exclusions from SDD occurred primarily at the pre-procedural assessment (49.4%), followed by post-procedure (43.8%) and intra-procedure assessments (6.8%). The most common cause for exclusion was related to conduction abnormalities (80.9%) (Fig. 2).

A greater proportion of female patients were suitable for SDD (55% vs. 21.5% *p* = 0.04). Otherwise, there were no demographic differences between the groups (Table 1). Procedural characteristics, including valve type, membranous septum length, annular measurements, replacement depth, and valve oversizing (%) were similar across both groups (Table 2).

**Table 1:** Baseline patient characteristics

	Same day discharge (*n* = 20)	Standard discharge (*n* = 162)	*p* value
Demographic Factors			
Age [mean, (SD)]	83.7 (6.7)	81.9 (6.4)	0.632
Sex (Female) [n (%)]	11 (55.0%)	51 (31.5%)	**0.036**
BMI [mean (SD)]	26.1 (5.0)	27.6 (5.4)	0.696
STS–PROM score [median, (IQR)]	3.1 (5.1)	2.5 (2.5)	0.140
Hypertension [n (%)]	16 (80.0%)	131 (80.9%)	0.926
Diabetes [n (%)]	4 (20.0%)	47 (29.0%)	0.397
Coronary Artery Disease [n (%)]	10 (50.0%)	51 (31.5%)	0.098
End-stage renal disease [n (%)]	1 (5.0%)	3 (1.9%)	0.368
Prior PCI [n (%)]	5 (25.0%)	42 (25.9%)	0.929
Prior CABG [n (%)]	2 (10.0%)	16 (9.9%)	0.986
Prior stroke [n (%)]	3 (15.0%)	13 (8.0%)	0.299
NYHA class (III or IV)	16 (80.0%)	136 (84.0%)	0.440
Mean aortic valve gradient (mmHg) (mean, SD)	42.9 (10.3)	45.1 (16.9)	0.278
Left ventricular ejection fraction (LVEF) (%) [mean (SD)]	59.0 (9.8)	58.7 (8.4)	0.912
Baseline ECG			
Atrial fibrillation [n (%)]	5 (25.0%)	22 (13.6%)	0.175
Sinus rhythm [n (%)]	13 (65.0%)	137 (84.6%)	**0.030**
First-degree AV block	0 (0%)	49 (30.2%)	-
Right bundle branch block [n (%)]	0 (0.0%)	31 (19.1%)	-
Left bundle branch block [n (%)]	1 (5.0%)	11 (6.8%)	0.761
Prior pacemaker [n (%)]	0 (0.0%)	0 (0.0%)	–

Bolded values indicate statistical significance (*p* < 0.05)

*BMI* Body mass index; *STS–PROM* Society of Thoracic Surgeons–Predicted Risk of Mortality; *PCI* percutaneous coronary intervention; *CABG* Coronary artery bypass graft; *NYHA* New York Heart Association; *AV* Atrioventricular

**Table 2:** Procedural characteristics

	Same day discharge (n = 20)	Standard discharge (n = 162)	*p* value
**Procedural characteristics**			
Valve type, n (%)			0.125
Self-Expanding	10 (50.0%)	109 (67.3%)	
Balloon-expandable	10 (50.0%)	53 (32.7%)	
Membranous septum length (mm) [mean, SD]	3.7 (1.8)	3.3 (2.4)	0.269
Annular measurements [mean, SD]			
Area	454.2 (118.7)	482.0 (101.3)	0.258
Perimeter	76.2 (9.9)	78.7 (8.2)	0.201
Valve oversizing% [mean, SD]	15.0 (9.7)	16.5 (8.6)	0.244
Replacement depth (mm) [mean, SD]	3.3 (2.0)	3.6 (2.0)	0.276

### Post-TAVR outcomes

No significant differences were found between SDD and standard discharge groups when comparing in-hospital and 30-day outcomes (Table 3). In the SDD cohort, there were 3 (15.0%) hospital readmissions within 30 days. One was a cardiovascular readmission (PPM on day 20, for complete heart block detected incidentally via loop recorder) and two non-cardiovascular readmissions (day 4 and day 22) (see Table 4 for further details). No other complications including stroke, bleeding, vascular site complications, delirium or myocardial infarction were recorded for the SDD cohort up to 30 days. The standard discharge cohort had 24 (14.8%) readmissions, of which 13 (8.0%) were cardiovascular and 11 (6.8%) were non-cardiovascular. The standard discharge cohort also reported 1 (0.6%) stroke, 1 (0.6%) major vascular complication, and 6 (3.7%) delayed pacemaker implantations after discharge and up to 30 days (Table 3).

**Table 3:** In hospital and 30-day mortality and morbidity outcomes for same day compared to standard discharge cohorts

	Same day discharge (n = 20)	Standard discharge (n = 162)	*p* value
In Hospital Outcomes			
Mortality, *n* (%)	0 (0.0%)	1 (0.6%)	0.890
Stroke, *n* (%)	0 (0.0%)	6 (3.7%)	0.492
Major Bleeding, *n* (%)	0 (0.0%)	4 (2.5%)	0.625
Vascular complications, *n* (%)	0 (0.0%)	7 (4.3%)	0.436
Permanent pacemaker implantation, *n* (%)	0 (0.0%)	29 (17.9%)	0.039
Delirium, *n* (%)	0 (0.0%)	4 (2.5%)	0.625
Myocardial infarction, *n* (%)	0 (0.0%)	0 (0.0%)	
Median time to discharge, days (IQR)	n/a	3.0 (2)	
Discharge to 30 day outcomes			
Mortality, *n* (%)	0 (0.0%)	0 (0.0%)	
Hospital readmission, *n* (%)	3 (15.0%)	24 (14.8%)	0.510
Cardiovascular hospitalisation	1 (5.0%)	13 (8.0%)	0.569
Non cardiovascular hospitalisation	2 (10.0%)	11 (6.8%)	0.346
Stroke, *n* (%)	0 (0.0%)	1 (0.6%)	0.890
Bleeding, *n* (%)	0 (0.0%)	0 (0.0%)	
V ascular complications, *n* (%)	0 (0.0%)	1 (0.6%)	0.890
Permanent pacemaker implantation, *n* (%)	1 (5.0%)	6 (3.7%)	0.508
Delirium, *n* (%)	0 (0.0%)	0 (0.0%)	
Myocardial infarction, *n* (%)	0 (0.0%)	0 (0.0%)

**Table 4:** Patients requiring any rehospitalisation in the SDD cohort (*n* = 3)

	Age (Sex)	BMI	STS%	Baseline bloods	Baseline TTE	Baseline ECG	Valve	Post-TAVR PVL	24 h ECG	Readmission (days post TAVR)	Reason for rehospitalisation
1	72 (F)	24.9	1.37	Hb 118 Cr 105	Mean gradient 41 mmHg LVEF 60%	SR PR: 140 ms QRS: 68 ms	BEV 20 mm	Trivial	SR PR: 152 ms QRS: 84 ms	20	Complete heart block detected via loop recorder
2	73 (M)	16.3	1.32	Hb 144 Cr 73	Mean gradient 44 mmHg LVEF 65%	AF QRS: 76	SEV 34 mm	Mild	AF QRS: 84 ms	4	Pneumonia
3	76 (F)	32.2	9.30	Hb 120 Cr 348	Mean gradient 28 mmHg LVEF 60% *low SVI	AF QRS: 71	SEV 26 mm	Trivial	AF QRS: 100 ms	22	Pneumonia

^*^low SVI (patient had paradoxical low flow low gradient severe aortic stenosis)

*BMI* Body mass index; *STS* Society of Thoracic Surgery Risk score; *TTE* Transthoracic echocardiogram; *PVL* Paravalvular leak; *TAVR* Transcatheter aortic valve replacement; *BEV* Balloon expandable valve; *SEV* Self-expandable valve; *Hb* Haemoglobin; *Cr* creatinine; *AF* Atrial fibrillation

The median time to discharge in the standard discharge cohort was 3 (± 2) days. No SDD patients experienced any adverse outcomes in the days between SDD (day 0) to the median time for non-SDD cohort discharge (day 3).

## Discussion

This study found that 11.0% of the studied cohort were eligible for SDD using the North Shore pathway. There were no deaths in the SDD group, and 30-day rehospitalisation rates were comparable to those of the standard discharge cohort.

The findings suggest that SDD would have been safe and feasible within this carefully selected low-risk TAVR cohort. The median length of stay in the standard discharge group was 3 days, and no complications were observed in the SDD group between day 0 and day 3, indicating no additional clinical benefit from keeping these select patients in hospital. The SDD cohort had a greater proportion of females but otherwise had no differences in demographic, imaging or procedural characteristics, compared to the standard discharge cohort.

Of the 182 patients screened, 20 (11.0%) were deemed suitable for SDD using the North Shore pathway, which differs to other major published studies. Barker et al. prospectively performed SDD in 5.9% of their cohort; whilst Krishnaswamy et al. retrospectively reported a 22.0% SDD rate during the COVID-19 pandemic. In this study, most exclusions for SDD occurred pre-procedurally (49.4%), followed by post-procedurally (43.8%) and intra-procedurally (6.8%). The most common exclusion reason was conduction-related (Fig. 2), accounting for 80.9% of cases and is an obstacle for widespread adoption of SDD. An important characteristic of our cohort was the absence of patients with a prior pacemaker, in accordance with the CONDUCT–TAVI protocol. In Barker et al.’s study, 32.3% of the SDD cohort had a pre-existing pacemaker, whilst 13.3% of the SDD cohort had a pacemaker in Krishnaswamy et al.’s cohort. Given the difficulty in reliably predicting post-TAVR conduction disease, patients with a prior pacemaker are likely to be the most ideal candidates for SDD.

Accurate risk stratification of patients at risk of delayed high-grade conduction abnormalities is essential for safe implementation of SDD. Conduction abnormalities after TAVR are primarily attributed to the anatomical proximity of the aortic valve complex to the membranous septum which harbours the Bundle of His [11]. Preexisting RBBB is an established predictor of post-TAVR pacemaker requirement and was a preprocedural exclusion criterion [12]. Persistent onset new LBBB occurs in 20% of patients post-TAVR and greatly increases the risk of delayed onset atrioventricular block after TAVR [13], and thus patients with new onset LBBB were also excluded from our SDD pathway. The incidence of delayed high-grade atrioventricular block is not well understood, with a recent systematic review estimating incidence at 5.2% [14]. Recent data showed the utility of post-TAVR invasive His-Ventricle (HV) interval > 70 ms to help further risk stratify patients at highest risk of complete heart block in post-TAVR LBBB [15], and this could be considered in future SDD pathways.

Self-expanding valves have been under-represented in prior SDD studies, making their safety and feasibility in SDD unclear. Prior studies conducted by Barker et al. and Krishnaswamy et al. used balloon expandable valves in 96.8% and 91.2% of their SDD cohorts, respectively. Although self-expanding valves have historically been an independent predictor of post-TAVR pacemaker requirement, advancements in deployment techniques, such as the adoption of the cusp-overlap technique [16] and higher depths of implantation, have resulted in a reduction in pacemaker rates with self-expanding platforms [17]. An important and unique strength of this paper is the use of self-expandable valves in 65.3% of the total cohort, supporting feasibility of SDD after TAVR irrespective of the valve choice.

Rehospitalizations rates were comparable between SDD and standard discharge cohorts, and where it occurred it was after standard discharge timeframes, supporting the safety of SDD. One cardiac re-hospitalisation in the SDD cohort occurred on day 20, relating to delayed onset complete heart block requiring PPM replacement. Our study patient with delayed PPM replacement had a balloon-expandable valve and was diagnosed as having complete heart block remotely from her loop recorder home monitoring and contacted to present to hospital. Two non-cardiovascular rehospitalisation occurred. All three rehospitalizations occurred after the median standard discharge time (3 days) as well as the individual patients’ discharge dates.

From a financial standpoint, it is estimated SDD could have enabled savings in our health district of up to $USD 8000 per patient, by preventing the median 3-night hospital admission ($USD 2670 per day). In this study cohort alone, SDD had a hypothetical cost-saving of $USD 160,000, as well as potentially allowing earlier treatment of 20 other patients due to additional bed availability. Prior Australian cost-effectiveness analyses on a low-risk TAVR cohort in 2021 have shown self-expanding valves to be economically dominant (compared to surgical aortic valve replacement), and balloon-expandable valves to be financially favourable, with an incremental cost-effectiveness ratio (ICER) of $AUD 3521 ($USD 2270) per quality adjusted life year compared to surgical aortic valve replacement [18]. Incorporating same-day discharge would equate to considerable economic and clinical dominance of TAVR, irrespective of valve choice, as a treatment for aortic stenosis.

### Limitations

As a retrospective sub-analysis of patients included in a separate prospective study cohort, our findings need dedicated and prospective validation. As a retrospective assessment, application of the North Shore clinical pathway was modified and approximated using clinical documentation, such as for mobility and vascular access site status, which may be prone to error. Furthermore, it must be noted that patients with a prior PPM were excluded from the original CONDUCT–TAVI study’s cohort. Although in this case, this may mean the present study underestimates the actual proportion of patients suitable for SDD. A prospective study of the North Shore pathway in an all-comer population is warranted and currently underway (ACTRN12624000571572).

## Conclusion

This study demonstrates that SDD after minimalist transfemoral TAVR can be safe and feasible in select low-risk patients, in both self-expanding and balloon-expandable valve cohorts. It also serves as a promising initial evaluation of the Australian derived North Shore Day Stay clinical pathway, which is currently undergoing prospective evaluation for use as a standardised protocol to guide SDD in clinical practice. SDD in selective patients may provide a strategy to streamline the accessibility of TAVR, improve hospital congestion and reduce costs amid growing demand for the procedure.

## Data Availability

Anonymised data related to the current study is available, subject to reasonable request to authors.
